# Habitual snoring and depressive symptoms during pregnancy

**DOI:** 10.1186/1471-2393-13-113

**Published:** 2013-05-16

**Authors:** Louise M O’Brien, Jocelynn T Owusu, Leslie M Swanson

**Affiliations:** 1 Sleep Disorders Center, Department of Neurology, University of Michigan, Ann Arbor, MI, USA; 2 Department of Oral and Maxillofacial Surgery, University of Michigan, Ann Arbor, MI, USA; 3 Sleep and Chronophysiology Laboratory, Department of Psychiatry, University of Michigan, Ann Arbor, MI, USA

**Keywords:** Pregnancy, Snoring, Sleep quality, Depressive symptoms, EPDS

## Abstract

**Background:**

Depression is frequently observed in patients with untreated sleep-disordered breathing (SDB) in the general population. Pregnant women are particularly vulnerable since pregnancy increases the risk of both SDB and depressive symptoms. However, no study has investigated whether SDB symptoms prior to or in early pregnancy are associated with such mood problems.

**Methods:**

A retrospective chart review of pregnant women. Women were included if they attended prenatal clinics between June 2007 and July 2010, were ≥18 years old, pregnant with a single fetus, and had been screened for habitual snoring as well as depressive symptoms using the Edinburgh Postnatal Depression Scales (EPDS).

**Results:**

In total, 362 women were included and 32.3% reported habitual snoring. Twenty-nine percent of women had an EPDS score ≥10. Significantly more snoring women, compared to non-snorers, had an EPDS score ≥10 (42.7% vs. 22.9%, p < 0.001) despite the mean EPDS values not reaching statistical significance (6.1 ± 4.9 vs. 5.4 ± 5.0, p = 0.2). In a logistic regression model controlling for parity, the presence of pre-pregnancy obesity, presence of a partner, sleep quality, African American race, maternal educational level, pre-eclampsia, and diabetes, snoring was independently associated with a prenatal EPDS score ≥10 (O.R. 2.0, 95%CI 1.13-3.46; p = 0.023).

**Conclusion:**

Maternal snoring may be a risk factor for prenatal depressive symptoms. Further investigation of the temporal relationship between maternal snoring and depressive symptoms is warranted.

## Background

Pregnancy is a time of increased vulnerability to affective illness [1]. The prevalence of depression in pregnancy is between 8% and 18% [1,2], with rates as high as 47-49% in minorities [3] and women of low socioeconomic status [1]. Prenatal depression is associated with poor birth outcomes [4], impaired maternal-infant bonding [5], and long-term consequences for mental health and development of offspring [6]. Despite the high prevalence of prenatal depression and its adverse consequences for mothers and infants, little is known about the pathogenesis and risk factors for depression during pregnancy, particularly risk factors that can be modified to improve outcomes for both women and infants.

Sleep disturbance is prevalent among pregnant women, particularly among depressed pregnant women [7]. Poor sleep quality in early pregnancy has been shown to predict increased depressive symptoms later in pregnancy both directly [8,9] as well as mediating the relationship between physical symptoms in early pregnancy and depressive symptoms in later pregnancy [10]. Furthermore, poor sleep quality during pregnancy is also associated with increased risk for postpartum depression [11]. Even after delivery, new mothers sleeping less than 4 hours per night are at increased risk for postpartum depression [12] and women with poor sleep quality in the initial postpartum weeks are at risk for recurrence of postpartum depression [13]. Indeed a complaint of trouble falling asleep may be one of the most relevant screening questions in relation to risk for postpartum depression [14]. In addition to the associations with depressive symptoms, sleep problems such as trouble falling/staying asleep or sleeping too much have been linked to poor health-related quality of life in pregnancy [15].

Nonetheless, no study of depressive symptoms in pregnancy has considered the presence of sleep-disordered breathing (SDB). Sleep-disordered breathing describes a spectrum of breathing disturbances during sleep, the major symptom of which is habitual snoring. Importantly, self reported depressive symptomatology is prevalent in SDB, with 20-30% of women attending a sleep clinic for SDB evaluation reporting a diagnosis of depression [16,17]. Moreover, in a recent study from the National Health and Nutrition Examination Survey of over 9,000 adults, snorting/stopping breathing at least 5 nights/week was associated with a 3-fold increased odds of probable major depression in women and a diagnosis of sleep apnea was associated with a 5-fold increased odds of probable major depression [18].

Pregnant women are at particular risk of SDB due to the changes in physiology that occur with pregnancy [19]. Accumulating data show that up to 35% of women in the 3rd trimester [20,21] and up to 85% of women with pre-eclampsia [22] habitually snore, likely due to weight gain, edema, and fluid shifts. Although the prevalence of polysomnographically-diagnosed SDB in pregnancy is currently unknown, recent data show that approximately 15% of obese women in the first trimester have obstructive sleep apnea [23], as do approximately half of women with gestational hypertension [24].

Although pregnant women are at increased risk of both SDB as well as mental health issues, no study has investigated a link between maternal SDB and depressive symptoms. As SDB may represent a modifiable risk for prenatal depression, it is crucial to understand associations between mood and SDB symptoms in pregnancy. This study aimed to determine whether there was preliminary evidence of a relationship between habitual snoring and prenatal depressive symptomatology.

## Methods

### Participants

These data comprise a retrospective review of medical records of women who had been previously screened for SDB symptoms during pregnancy [21] between July 2007 and July 2010 and who had also been clinically screened for depressive symptoms. Clinical screening for depressive symptoms, using the Edinburgh Postnatal Depression Scale (EPDS), is part of routine clinical care at our institution. The study was approved by the University of Michigan Institutional Review Board. Since this study was a retrospective review of data already prospectively obtained, informed consent was not obtained from subjects.

### Data collection

Sleep data retrospectively extracted for the current study were originally obtained from women during the 3^rd^ trimester of pregnancy. This included the presence of habitual snoring and “stopped breathing/gasped for air” at night [21]. Habitual snoring had been defined as snoring either “3-4 times per week” or “almost everyday”. The timing of snoring had also been noted which allowed classification into controls (non-snorers both before pregnancy and during the pregnancy through the 3^rd^ trimester) and chronic snorers (snorers prior to pregnancy or those who started habitually snoring before 28 weeks’ gestation). In addition to SDB symptoms, self-reported bed times and wake times had been also obtained and sleep duration calculated. In the current study short sleep duration was defined as self-reported sleep duration of ≤6 hours per night and long sleep duration was defined as self-reported sleep duration ≥10 hours per night. Sleep quality and daytime function had been assessed using the 21-item self-report General Sleep Disturbance Scale (GSDS) [25]. Women had been asked to provide the frequency of specific sleep complaints from 0 (not at all) to 7 (every day). The GSDS comprises several subscale domains including sleep quality and daytime function. Consistent with the Diagnostic and Statistical Manual of Mental Disorders criteria for insomnia (American Psychiatric Association 1994), clinically significant sleep disturbance was identified by a mean domain score of 3 or more.

### Depressive symptoms

The EPDS is a 10-item self-report, depressive symptoms screening questionnaire that has been validated for the identification of depressive symptoms over the previous 7 days in pregnant and postpartum women [26]. Scores range from 0 to 30; higher scores indicate more depressive symptoms. A threshold of 9/10 has been suggested as an appropriate threshold for routine use in primary care to identify women with depressed mood [26,27]. Thus, in the present study women with a score ≥10 were classified as having depressive symptoms. However, it has also been suggested that a higher threshold (e.g. EPDS ≥ 15) may be more appropriate for pregnant women due to the heightened anxiety during gestation [27]; analyses were therefore repeated for this subgroup.

### Other variables

Other variables extracted from medical records included demographics (age, race, educational level, marital status), height, weight, and parity. Obesity was defined as a pre-pregnancy Body Mass Index (BMI) ≥30 kg/m^2^. Data were also collected on previous history of depressive disorder, current diagnosis of depressive disorder, previous or family history of gestational hypertension or pre-eclampsia, smoking status, a diagnosis of chronic hypertension, gestational hypertension, pre-eclampsia, or gestational diabetes. The latter diagnoses were obtained from medical coding using the International Classification of Diseases, 9th edition (ICD-9) [28] and were verified using medical records.

### Statistics

All data obtained were double-entered into a database and analyzed with SPSS (version 19.0, IBM). Histograms, box-plots, and descriptive methods were used to examine data for errors and outliers. Between-group comparisons of continuous variables (EPDS score, maternal age, BMI, and gestational age) were conducted with t-tests (snoring vs. no snoring). Dichotomized variables (positive screen for depressive symptoms vs. negative screen for depressive symptoms) were compared with Chi Square tests. Correlations between continuous variables (EPDS score, sleep duration, sleep quality score, and daytime function score) were conducted using Pearson’s Correlation Coefficient. Logistic regression was used to determine associations between snoring and depressive symptoms after adjusting for potential covariates where appropriate (including maternal race, pre-pregnancy obesity, parity, educational level, presence of a partner, pre-eclampsia, diabetes, sleep quality score). Odds ratios (OR) and 95% Confidence Intervals (CI) were calculated. A p-value <0.05 was considered statistically significant.

## Results

In total, 373 pregnant women who were screened for depressive symptoms with the EPDS were also screened for symptoms of SDB. Women were included in this analysis if they reported non-snoring prior to pregnancy *and* non-snoring in the 3^rd^ trimester (n = 245) or if they reported chronic/early pregnancy snoring (n = 117). Women who reported snoring onset only during the 3^rd^ trimester were excluded (n = 11) since the depression screen clearly predated the onset of snoring. Therefore, the following analyses were based on a sample size of n = 362.

The majority of depression screens occurred prior to 28 weeks gestation (90.6%) with the mean gestation at screening being 13.6 ± 8.2 weeks. Exclusion of the 9.4% of women who completed the depression screen after 28 weeks gestation did not change our findings (data not shown) and therefore all n = 362 women remained in the analyses. Table 1 shows the demographics of the sample.

**Table 1:** Demographic data

	**Total sample (n = 362)**
Age (years)	30.1 ± 5.9
Pre-pregnancy BMI (kg/m^2^)	27.0 ± 7.3
Obesity (BMI ≥ 30;%)	27.1%
Racial Background:	
Caucasian (%)	72.9%
African American (%)	12.2%
Asian (%)	8.9%
Multi-racial/Other (%)	6.1%
Educational Level:	
High school or less (%)	24.5%
Marital Status:	
Married (%)	67.7%
Partner (%)	3.9%
Single (%)	22.9%
Separated (%)	0.8%
Divorced (%)	0.6%
Unknown (%)	4.1%
Nulliparous (%)	32.0%
Pre-eclampsia (%)	7.8%
Diabetes Mellitus (%)	14.6%
Smoker (%)	14.5%

Data shown as mean ± standard deviation, or proportion as appropriate.

BMI = Body Mass Index.

A total of 106 women (29.3%) were found to have an EPDS score ≥10. African American women were more likely than others to have depressive symptoms (45.5% vs. 27.0%, p = 0.02); similarly, women who were obese prior to pregnancy were more likely to have EPDS scores ≥10 compared to non-obese women (41.8% vs. 25.2%, p = 0.004). Women who had given birth before were more likely than those who had never given birth to have EPDS scores ≥10 (33.8% vs. 21.2%, p = 0.02). See Table 2 for differences in demographic and sleep variables between women with and without EPDS scores ≥10.

**Table 2:** Comparison of demographics and sleep variables in women with and without EPDS scores ≥10

	**EPDS < 10 (n = 256)**	**EPDS ≥ 10 (n = 106)**
Age (years)	30.3 ± 5.8	29.5 ± 5.8
Pre-pregnancy BMI (kg/m^2^)	26.0 ± 6.6	29.1 ± 8.2**
Obesity (BMI ≥ 30;%)	22.0%	38.7%**
African American (%)	9.2%	18.3%*
High school or less (%)	20.6%	32.1%*
Married/partnered (%)	81.2%	59.2%**
Nulliparous (%)	36.1%	22.6%*
Pre-eclampsia (%)	5.2%	14.0%**
Diabetes Mellitus (%)	14.6%	15.0%
Smoker (%)	8.4%	25.0%**
Snoring (%)	26.2%	47.1%
Stopped breathing (%)	4.1%	3.8%
Mean Sleep Duration (hours)	8.7 ± 1.4	8.6 ± 1.8
Sleep duration ≤6 hours (%)	2.8%	7.3%
Sleep duration >10 hours (%)	13.3%	11.8%
Mean Sleep quality score	3.5 ± 1.2	4.1 ± 1.1**
Poor sleep quality (%)	67.8%	83.8%**

*p < 0.05; **p < 0.001.

BMI = Body Mass Index.

EPDS = Edinburgh Postnatal Depression Scale.

Significantly more snoring women, compared to non-snorers, had an EPDS score ≥10 (42.7% vs. 22.9%, p < 0.001) despite the mean EPDS values not reaching statistical significance (6.1 ± 4.9 vs. 5.4 ± 5.0, p = 0.25). Only 15 women reported that they stopped breathing or gasped for air. The proportion of women in this group who had EPDS scores ≥10 was similar to the proportion of women who did not report that they stopped breathing (26.6 vs. 27.9%, p = 1.0). Furthermore, their mean EPDS scores were not different from those who did not endorse this symptom (4.9 ± 4.5 vs. 5.3 ± 4.8, p = 0.79).

When using a threshold EPDS score ≥15, similar findings were observed. African American women were more likely than others to have EPDS ≥15 (24.4% vs. 13.1%; 0 = 0.06), as were obese women (23.2% vs. 11.4%, p = 0.007). There was a tendency for snoring women, compared to non-snorers, to have EPDS scores ≥15 although this did not quite reach statistical significance (18.3% vs. 12.8%; p = 0.19). In the subgroup of women with EPDS scores ≥15, 43.6% were snorers compared to 25.9% of women whose EPDS scores were <9 (p = 0.015).

Demographic variables together with the mean EPDS scores, sleep durations, and sleep quality scores for snoring women and non-snoring women are shown in Table 3. Only a minority of women (4.1%) had short sleep duration (≤6 hours) while 12.0% had long sleep duration (≥10 hours). Sleep duration was not correlated with sleep quality (r = −0.01, p = 0.85) or daytime function (r = 0.03, p = 0.59). Neither was there a relationship between self-reported sleep duration during pregnancy and EPDS scores (r = −0.02, p = 0.73). Although more women with short sleep duration, compared to those with >6 and <10 hours of sleep duration, reported depressive symptoms, this was not statistically significant (50.0% vs. 26.1%; p = 0.21). A similar proportion of women with long sleep duration, compared to those with >6 and <10 hours, reported depressive symptoms (25.3 vs. 26.1, p = 1.0).

**Table 3:** Comparison of demographics, mean EPDS, sleep duration, and sleep quality scores between non-snoring and snoring women

	**Non-snoring (n = 245)**	**Snoring (n = 116)**
Age (years)	29.7 ± 5.8	30.8 ± 6.0
Pre-pregnancy BMI (kg/m^2^)	25.4 ± 6.1	30.4 ± 8.4**
Obesity (BMI ≥ 30;%)	19.7%	43.0%**
African American (%)	10.7%	14.8%
High school or less (%)	22.5%	25.2%
Married/partnered (%)	78.4%	67.3%*
Nulliparous (%)	35.4%	24.8%*
Pre-eclampsia (%)	6.2%	11.3%
Diabetes Mellitus (%)	11.6%	20.9%*
Smoker (%)	8.5%	21.9%**
Stopped breathing (%)	1.7%	9.6%**
Mean EPDS	5.4 ± 5.0	6.1 ± 4.9
EPDS ≥10 (%)	22.9%	42.7%**
EPDS ≥15 (%)	12.8%	18.3%
Mean Sleep Duration (hours)	8.7 ± 1.6	8.7 ± 1.6
Sleep duration ≤6 hours (%)	5.1%	1.9%
Sleep duration >10 hours (%)	12.6%	14.9%
Mean Sleep quality score	3.5 ± 1.2	3.9 ± 1.2*
Poor sleep quality (%)	72.5%	74.6%**

*p < 0.05; **p < 0.001.

BMI = Body Mass Index.

EPDS = Edinburgh Postnatal Depression Scale.

Both sleep quality and daytime function scores showed weak-to-moderate positive correlation with EPDS scores (r = 0.24 and r = 0.39, p < 0.001 respectively). Women with poor sleep quality (domain score ≥3), compared to those without, were more likely to have an EPDS score ≥10 (33.5% vs. 17.5%; p = 0.004). Similarly, women with poor daytime function (domain score ≥3) were also more likely to have EPDS scores ≥10 (35.7% vs. 10.6%; p < 0.001).

In a logistic regression controlling for parity, the presence of pre-pregnancy obesity, presence of a partner, sleep quality score, African American race, maternal educational level (high school or less), pre-eclampsia, and diabetes, snoring was independently associated with a prenatal EPDS score ≥10 (O.R. 2.0, 95%CI 1.13-3.46; p = 0.023). This model accounted for 24.2% of the variance in EPDS score. The regression model is shown in Table 4.

**Table 4:** Logistic regression of EPDS ≥ 10 onto snoring and other covariates

**Explanatory variables**					
**Variable**	**Beta**	**SE**	**p-value**	**Adjusted OR**	**95%CI**
Snoring	0.666	0.293	0.023	2.00	1.13 – 3.46
Parity	0.273	0.122	0.025	1.38	1.04 – 1.67
Obesity	0.433	0.320	0.177	1.54	0.82 – 2.89
Sleep quality score	0.456	0.127	0.001	1.58	1.23 – 2.03
Partner	−0.802	0.348	0.021	0.45	0.23 – 0.89
African American	0.096	0.432	0.824	1.10	0.47 – 2.57
High school or less	−0.400	0.375	0.286	0.67	0.32 – 1.40
Pre-eclampsia (%)	1.552	0.478	0.001	4.72	1.85 – 12.05
Smoker (%)	0.969	0.427	0.017	2.64	1.19 – 5.83

EPDS = Edinburgh Postnatal Depression Scale.

SE = standard error around the coefficient for the constant.

OR = odds ratio.

CI = confidence interval.

## Discussion

These novel findings suggest that habitual snoring which began prior to or in early pregnancy is associated with prenatal depressive symptoms even after controlling for the known relationship between poor sleep quality and depression. These data raise an important issue about the potential role of habitual snoring, as a marker for SDB, in mood disorders during pregnancy and subsequently the development of postpartum depression.

In non-pregnant adults, the association between SDB and depressive symptoms has received considerable attention [18,29]. In recent years several studies have shown a relationship between poor sleep and depressive symptoms in pregnant and postpartum women [9-11,14,30-32]. In a prospective study of 240 pregnant women in the second trimester, of which 59 were depressed, Okun et al. [7] found that depressed women had more fragmented sleep as reflected by longer sleep latencies, longer periods of nocturnal wakefulness, and poorer sleep efficiency than non-depressed women. In addition, in the non-depressed women those with short or longer sleep durations, symptoms of insomnia, and long periods of nocturnal wakefulness had higher scores on a depression rating scale. Data from the present study further support the relationship between sleep quality and depressive symptoms, but we did not find a relationship between sleep duration and EPDS score. However, few women in our study had short sleep and, unlike Okun et al., [7] we were unable to assess any longitudinal relationship due to the retrospective design.

In addition to a direct relationship between sleep disruption and depressive symptoms, sleep quality has also been shown to mediate the relationship between early pregnancy-related physical symptoms and later depressive symptoms [10]. Recently, a hypothesis was proposed illustrating how poor sleep quality and sleep deprivation during pregnancy could lead to a negative impact on the mother-infant relationship [33]. Nonetheless, despite a growing literature describing the role of poor sleep quality and sleep deprivation in pre- and postpartum depressive symptomatology, no previous study has included SDB. This is notable, since approximately 15% of first trimester pregnant women have SDB [23] and the proportion of women with SDB symptoms or diagnosed SDB is considerably increased in later stages of pregnancy [20,21,34]. Understanding the role of SDB in mood disorders in pregnant women is therefore of critical importance. Sleep disordered breathing is associated with overweight and obesity and the proportion of women of childbearing age who are overweight has significantly increased in recent decades [35].

Understanding modifiable key risk factors for prenatal depression is of tremendous importance for public health, since depression is often missed during the prenatal period, [36] yet it is a significant predictor of postpartum depression [37]. Interventions implemented during the prenatal period may be an effective strategy for prevention of postpartum depression [38]. Untreated prenatal depression is associated with a host of negative outcomes for women and their children, including participation in unhealthy practices such as smoking, alcohol use, and drug abuse [39], poor birth outcomes [4], impaired maternal-infant bonding [40,41], increased risk for developmental delays [6], and mental illness [42]. The most catastrophic outcome of prenatal depression is suicide. A recent systematic review identified life stress, lack of social support, and domestic violence as major risk factors for prenatal depression [43]. While largely modifiable, significant resources are required to address such factors. The results from the present study suggest that SDB, a common condition in pregnancy [20,21,44,45] is a possible risk factor for prenatal depression. Importantly, SDB can be treated.

In this study we chose a threshold for depressive symptoms to be an EPDS score ≥10. While this is a recognized, validated, and commonly used threshold in the postpartum period, and also during the prenatal period at our institution, it is possible that a higher threshold should be used in prenatal women. Indeed a threshold of ≥15 has been suggested for pregnant women [27]. The overall number of women with these higher EPDS scores was small but our findings were similar to those using an EPDS score ≥10. Notably, almost half of women with EPDS scores ≥15 reported chronic/early pregnancy snoring. This further supports a role for SDB in depressive symptoms in pregnant women.

Several limitations of the current study should be considered. The main limitation is that the temporal relationship between snoring and depressive symptoms cannot be confirmed in a retrospective study. This design precluded the collection of depressive measures prior to pregnancy thus we were unable to measure the true incidence of depressive symptomatology during pregnancy. Nonetheless, this did not prevent investigation of the associations with snoring. In addition, it is possible that women did not accurately recall the presence of habitual snoring prior to pregnancy. However, the frequency of habitual snoring prior to pregnancy is similar to that reported in non-pregnant women of childbearing age or women in the early stages of pregnancy [21,34,46] thus any recall bias is likely minimal. We did not find a relationship between reports of stopping breathing/gasping for air and depressive symptoms. While habitual snoring is the hallmark symptom of SDB, “stopping breathing” is also an important symptom of SDB. However, the number of women who endorsed this symptom was small and therefore limits the conclusions that can be drawn. Finally, no objective measures of sleep were used in the present study. While a combination of actigraphy and polysomnography would have provided objective evidence of sleep duration, sleep fragmentation, and severity of SDB, physiological monitoring is not logistically or financially possible in a large cohort of women. However, self-report of snoring is a reasonable predictor of objective evidence of SDB on polysomnography [47]. Moreover, subjective perception of sleep difficulties, such as sleep quality, is often predictive of poor outcomes [48-51] yet is not captured by objective assessments. Thus, the lack of objective sleep data is unlikely to significantly impact our findings.

## Conclusions

In summary, maternal snoring appears to be independently associated with prenatal depressive symptoms as measured by the EPDS. These findings are important particularly since snoring affects a large number of pregnant women, increases in frequency as gestation progresses, and prenatal depressive symptoms remain one of the most common and consequential conditions during pregnancy. While the temporal relationship between snoring and depressive symptoms remains to be confirmed, these novel data suggest that routine screening for SDB symptoms during pregnancy may have clinical utility in early identification of women at risk for depressive symptoms and may provide an opportunity for intervention.

## Abbreviations

SDB: Sleep-Disordered Breathing; EPDS: Edinburgh Postnatal Depression Scale; GSDS: General Sleep Disturbance Scale; BMI: Body Mass Index; ICD: International Classification of Diseases.

## Competing interests

The authors declare that they have no competing interests.

## Authors’ contributions

LMO conceived and designed the study, assisted with data acquisition, analyzed and interpreted the data, drafted the manuscript, and approved the final version for submission. JTO assisted with study design, conducted data acquisition, managed, checked, and cleaned the data, assisted with manuscript editing, and approved the final version. LMS assisted with study design, analysis and interpretation as well as participated in manuscript drafting and revision, and provided approval for the final submitted version. All authors read and approved the final manuscript.

## Pre-publication history

The pre-publication history for this paper can be accessed here:

http://www.biomedcentral.com/1471-2393/13/113/prepub
